# Detection of Novel Coronavirus by RT-PCR in Stool Specimen from Asymptomatic Child, China

**DOI:** 10.3201/eid2606.200301

**Published:** 2020-06

**Authors:** An Tang, Zhen-dong Tong, Hong-ling Wang, Ya-xin Dai, Ke-feng Li, Jie-nan Liu, Wen-jie Wu, Chen Yuan, Meng-lu Yu, Peng Li, Jian-bo Yan

**Affiliations:** Zhoushan Center for Disease Control and Prevention, Zhoushan, China

**Keywords:** coronavirus, novel coronavirus, 2019 novel coronavirus disease, COVID-19, severe acute respiratory syndrome coronavirus 2, SARS-CoV-2, viruses, respiratory infections, reverse transcription PCR, RT-PCR, stool, asymptomatic child, China

## Abstract

We report an asymptomatic child who was positive for a coronavirus by reverse transcription PCR in a stool specimen 17 days after the last virus exposure. The child was virus positive in stool specimens for at least an additional 9 days. Respiratory tract specimens were negative by reverse transcription PCR.

An outbreak of coronavirus disease (COVID-19) began in Wuhan, China, during December 2019 and has rapidly spread throughout China and to many countries (*1*,*2*). Common symptoms include fever, dry cough, and myalgia (*3*). Ten laboratory-confirmed cases and several asymptomatic cases of COVID-19 have been identified in Zhoushan, China, since January 19, 2020. We report the epidemiologic and diagnostic features for 1 case in an asymptomatic child.

On January 30, 2020, we identified a 10-year-old boy who had no fever or cough but had close contact with 2 confirmed case-patients with laboratory-confirmed COVID-19 (Figure). The boy was a primary school student who lived with his parents in an apartment of a college. The complex had several confirmed COVID-19 case-patients during January 19–31. 

**Figure:**
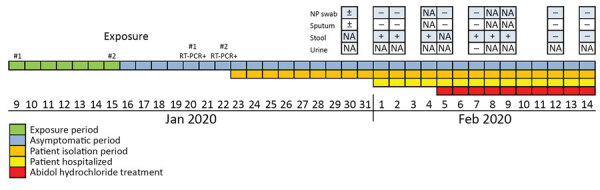
Timeline for detection of novel coronavirus by RT-PCR in stool specimen from asymptomatic child, China, January 9–February 14, 2020. NA, not available; NP, nasopharyngeal; RT-PCR, reverse transcription PCR; +, positive for novel coronavirus RNA by RT-PCR; ±, equivocal for novel coronavirus RNA by RT-PCR; –, negative for novel coronavirus RNA by RT-PCR.

Interviews of the boy and his parents confirmed his multiple exposures to the previously confirmed case-patients. On January 9 and 15, he participated in 2 parties with his parents and their colleagues. Two persons at these parties were positive for severe acute respiratory syndrome coronavirus 2 (SARS-CoV-2) by reverse transcription PCR (RT-PCR) on January 20 and 22. During January 12–15, the boy played football at a football club with a teammate who had a virus-positive RT-PCR result on January 22. The parents of the boy were asymptomatic and their stool, nasopharyngeal, and sputum specimens collected on February 1 and 14 were negative for SARS-CoV-2.

We collected nasopharyngeal swab and sputum samples from the boy 15 days after the last close contact and tested these specimens for SARS-CoV-2 by using RT-PCRs targeting the open reading frame lab (ORF1ab) and nucleoprotein gene regions (*4*). We obtained equivocal results: cycle threshold (C_t_) values were negative for ORFlab and 37.5 for the nucleoprotein gene. However, on February 1 (17 days after his last contact), a stool specimen was positive for SARS-CoV-2 by RT-PCR. (ORF1ab C_t_ 32.6; nucleoprotein gene C_t_ 33.7). He was then hospitalized in isolation and for monitoring.

Since January 22, The area of residence for the boy had been isolated, and community physicians monitored quarantined residents twice a day for signs and symptoms including fever, cough, and myalgia. During January 9–31, the boy had no signs or symptoms.

In the hospital, a routine blood test performed on February 2 showed cell counts within reference ranges, and a computed tomography scan on February 5 showed no abnormalities. After additional stool specimens collected on February 2 (ORF1ab C_t_ 25.6; nucleoprotein gene C_t_ 25.8) and February 4 (ORF1ab C_t_ 25.6; nucleoprotein gene C_t_ 28.3) were positive, the patient received abidol hydrochloride (100 mg 3×/d), interferon α-2b spray (2.5 million U 2×/d) and traditional Chinese medical therapy on February 5. Stool specimens collected on February 7 (ORF1ab C_t_ 26.3; nucleoprotein gene C_t_ 27.6), February 8 (ORF1ab C_t_ 31.4; nucleoprotein gene C_t_ 30.6), and February 9 (ORF1ab C_t_ 27.0; nucleoprotein gene C_t_ 27.0) were positive, but stool specimens collected on February 12 and 14 were negative.

Early symptoms in most COVID-19 patients include fever, myalgia, cough, and sore throat (*5*), which are common in other acute respiratory virus infections (*6*). Most cases appear to be mild, and most hospitalized patients have pneumonia with ground glass opacities on chest radiographs. Few children with SARS-CoV-2 infections have been reported, and most of them had mild clinical symptoms (*7*).

The boy we report had close contact with confirmed COVID-19 case-patients on several occasions before he showed an equivocal RT-PCR result for respiratory specimens and subsequently positive results for stool specimens. Despite these positive test results, he had no detectable fever or other clinical symptoms consistent with COVID-19 for >30 days from his last documented exposure. Although positive RT-PCR results do not necessarily indicate presence of infectious virus, our findings reinforce the need for RT-PCR testing of asymptomatic persons with exposure to COVID-19 patients. Asymptomatic infections complicate efforts to curtail SARS-CoV-2 transmission and implement effective control procedures.

SARS-CoV-2 is believed to be transmitted through large respiratory droplets (*8*) and close contact (*9*). Indirect transmission by contaminated fomites might also play a role. During the SARS pandemic of 2002–2003, positive RT-PCR results for stool specimens from SARS patients suggested that stools or sewage might be virus sources (*10*). Our finding of multiple positive stool specimens in this case similarly raises the concern that stool from COVID-19 patients might serve as another vehicle for virus transmission. Moreover, detection of virus by RT-PCR in stool specimens when respiratory tract specimens are negative suggests that stool might be considered, in addition to respiratory tract specimens, for routine diagnostic screening.

Our study had several limitations. The delay in RT-PCR testing after the first recognition of virus exposure prevented a more accurate estimation of the incubation time from exposure to RT-PCR positivity. The failure to test other specimens, such as blood and urine, prevented determination of the full spectrum of virus shedding for the case-patient. Although we urge caution in making policy decisions on the basis of 1 case, expanded testing of various clinical specimens from symptomatic and asymptomatic case-patient contacts at multiple time points would be warranted to help confirm our findings.
